# High secondary attack rate and persistence of SARS-CoV-2 antibodies in household transmission study participants, Finland 2020–2021

**DOI:** 10.3389/fmed.2022.876532

**Published:** 2022-07-28

**Authors:** Timothée Dub, Anna Solastie, Lotta Hagberg, Oona Liedes, Hanna Nohynek, Anu Haveri, Camilla Virta, Saimi Vara, Mervi Lasander, Nina Ekström, Pamela Österlund, Katja Lind, Hanna Valtonen, Heidi Hemmilä, Niina Ikonen, Timo Lukkarinen, Arto A. Palmu, Merit Melin

**Affiliations:** ^1^Infectious Disease Control and Vaccinations Unit, Department of Health Security, Finnish Institute for Health and Welfare (THL), Helsinki, Finland; ^2^Expert Microbiology Unit, Department of Health Security, Finnish Institute for Health and Welfare (THL), Helsinki, Finland; ^3^Centre for Military Medicine, Finnish Defence Forces, Helsinki, Finland; ^4^Health Stations and Internal Medicine Clinic, Social and Health Care Sector, Helsinki, Finland; ^5^Department of Public Health Solutions, Finnish Institute for Health and Welfare, Tampere, Finland

**Keywords:** COVID-19, household transmission, neutralizing antibodies, secondary attack rate, antibody persistence

## Abstract

**Background:**

Household transmission studies offer the opportunity to assess both secondary attack rate (SAR) and persistence of SARS-CoV-2 antibodies over time.

**Methods:**

In Spring 2020, we invited confirmed COVID-19 cases and their household members to four visits, where we collected nasopharyngeal and serum samples over 28 days after index case onset. We calculated SAR based on the presence of SARS-CoV-2 neutralizing antibodies (NAb) and assessed the persistence of NAb and IgG antibodies (Ab) against SARS-CoV-2 spike glycoprotein and nucleoprotein.

**Results:**

SAR was 45% (39/87), including 35 symptomatic secondary cases. During the initial 28-day follow-up, 62% (80/129) of participants developed NAb. Of those that seroconverted, 90% (63/70), 85% (63/74), and 78% (45/58) still had NAb to early B-lineage SARS-CoV-2 3, 6, and 12 months after the onset of the index case. Anti-spike IgG Ab persisted in 100% (69/69), 97% (72/74), and 93% (55/59) of seroconverted participants after 3, 6, and 12 months, while anti-nucleoprotein IgG Ab levels waned faster, persisting in 99% (68/69), 78% (58/74), and 55% (39/71) of participants, respectively.

**Conclusion:**

Following detection of a COVID-19 case in a household, other members had a high risk of becoming infected. NAb to early B-lineage SARS-CoV-2 persisted for at least a year in most cases.

## Introduction

In December 2019, SARS-CoV-2, a novel coronavirus causing COVID-19 was detected in Wuhan, China (1). Its emergence rapidly led to a pandemic and more than 500 million confirmed cases, including six million deaths, have been reported (2, 3). As with any newly detected respiratory pathogen, the emergence of SARS-CoV-2 was associated with a lack of knowledge of several key parameters in infectious disease epidemiology, including transmission dynamics, the expected severity of the infection, the development of antibodies and duration of immunity.

As a household is a closed setting with a well-defined population, implementing household transmission studies can be a pragmatic approach to assess viral transmission patterns at an early stage of an epidemic (4). Such studies can also provide better insight on the spectrum of disease, while initial surveillance focuses and detects primarily the most severe cases of emergent pathogens, giving a skewed clinical picture of the emerging disease.

In Finland, the first locally acquired case was detected in February 2020 and public health measures to control transmission were first implemented on March 12, 2020 (5). At this time, knowledge of transmission dynamics was still limited, and according to the WHO China Joint Mission, most clusters had occurred within families and the secondary attack rate was estimated to range between 3 and 10% in household settings (6). In order to participate in the international effort on improving knowledge of SARS-CoV-2 transmission patterns, we undertook a SARS-CoV-2 household transmission study in the Helsinki capital area and followed study participants for 12 months to assess the long-term persistence of SARS-CoV-2 specific antibodies (Ab).

## Materials and methods

### Study design and participants

We conducted a household transmission study starting in March 2020 using the Household transmission investigation protocol developed by the World Health Organization (4). We invited a convenience sample of cases with a recent PCR-confirmed SARS-CoV-2 infection and their household members residing in the Helsinki capital area to participate in four household visits on 0, 7, 14, and day 28 after the onset of the index case. Participants were either recruited after an invitation by a text message on a phone number obtained during the contact enquiry of the index case or COVID-19 cases could directly be enrolled after reading the study information on the institute’s website (7). Participants living by themselves, i.e., not in a household and participants living in residential institutions, dormitories, hotels/hostels or shelters were not eligible to participate.

At each of the four visits, we collected blood (venous sample in most cases, fingertip when not possible) and nasopharyngeal samples of all index cases and household members to detect infections by PCR and to assess SARS-CoV-2 Ab induced by infection. We also collected data from index cases and contacts on symptoms and their date of onset, as well as a symptom diary collecting the history of symptoms and their onset for 28 days following the onset of the index case.

We invited cases that had developed NAb by day 28 visit for serum collection at 3 months post-onset of the index case and all participants for serum collection at 6 and 12 months post-onset of the index case. Participants that would have received a COVID-19 vaccine between the 6 and 12 months sample collections were included in the assessment of the persistence of anti-nucleoprotein IgG Ab but excluded from the assessment of NAb and anti-spike IgG Ab persistence at 12 months.

### Laboratory analyses

We extracted RNA from nasopharyngeal specimens using the Qiagen QiacubeR instrument with RNeasy Mini KitR (QIAGEN, United States). We synthesized cDNA using random hexamer primers and RevertAid H Minus Reverse Transcriptase (Thermo Scientific, United States) and ran RT-PCR tests using QuantiTect™ Multiplex NoRox PCR Kit (QIAGEN, United States). We used previously described (8) primers and probes.

To measure SARS-CoV-2-specific NAb, we used the cytopathic effect (CPE)-based microneutralization test as previously described (9, 10) against two SARS-CoV-2 isolates representing B lineage (hCoV-19/Finland/1/2020 (GISAID accession ID EPI_ISL_407079): Fin1-20 and B.1 lineage hCoV-19/Finland/FIN-25/2020 (EPI_ISL_412971): Fin25-20.). A sample was considered negative if its NAb titer for both viruses was < 4, and positive if its titer was > 4 for at least one virus. If the titer for both viruses was 4, the sample was considered borderline. Samples with negative titers were given a value of 2 for statistical analysis.

We measured IgG to SARS-CoV-2 with a previously described in-house fluorescent multiplex immunoassay, which quantifies IgG Ab to SARS-CoV-2 nucleoprotein, SFL and RBD simultaneously (10, 11). Participants that received COVID-19 vaccine between 6- and 12-month sample collections were included in the assessment of the persistence of anti-nucleoprotein IgG Ab but excluded from the assessment of NAb and anti-spike IgG Ab persistence at 12 months.

### Operational definitions

We defined a household as a group of two people or more living in a domestic residence and a household contact as a person who resided in the same household as the COVID-19 index case while symptomatic. For inclusion of a household, at least one member had to have a recently confirmed SARS-CoV-2 infection by RT-PCR through a routine healthcare contact. This household member was considered the index case. We defined a case as a participant who either had RT-PCR confirmation of SARS-CoV-2 infection or who had developed NAb by the end of the initial follow-up.

Index cases, i.e., “the first household member receiving a positive diagnosis” were considered as primary cases, i.e., “the person who would bring a disease into a group” (12) in households where the index case was the first one to have experienced symptoms. If a household contact reported earlier symptom onset than the index case, then they were reclassified as the primary case and the index case as a secondary case. If the onset of symptoms was the same for the index and at least one household secondary case, then this household was excluded from the household transmission analysis but still included in the long-term serological follow-up.

### Statistical analysis

We calculated the number of secondary cases overall and per household as well as the effective household reproduction number depending on the age of the primary case. We described age, sex, pre-existing conditions, and symptoms of the healthy household members and secondary cases and compared them using Chi2 or Fischer exact test when sample size required. Geometric mean concentrations (GMC), titers (GMT), and their 95% confidence intervals (CIs) were used in the assessment of antibody trends among participants that had had asymptomatic, mild and severe symptoms. We also assessed the persistence of antibodies in the initially seropositive cases over a 12-month period. We performed statistical analysis using STATA 15.1 (StataCorp LP Lakeway, TX, United States).

## Results

### Household transmission

**FIGURE 1:**
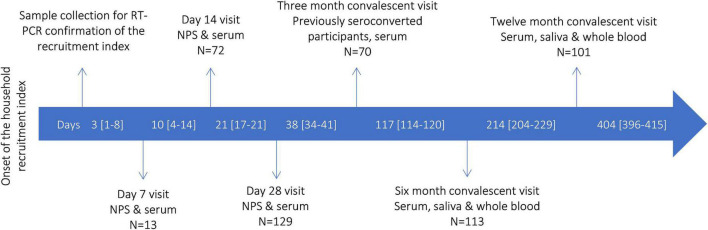
SARS-CoV-2 household transmission, Finland 2020–2021: study design and timeline of recruitment and follow-up (N total, 129 participants), median days [interquartile range] passed from the onset of the index case. NPS, nasopharyngeal specimen collection.

Between March 24th and June 17th 2020, we recruited 39 index cases and 90 household members, i.e., a total of 129 participants from 39 households. Due to restricted access to RT-PCR testing for SARS-CoV-2 and delayed availability of test results during the early months of the pandemic, participants were recruited, on average, 26 days [median, interquartile range (IQR): 19–40] after symptom onset of the index case. Hence only four households were visited around days 7, 14, and 28; 13 households were visited around 14 and day 28, while in 20 households, only one visit was conducted. On average, the last household transmission study visit was conducted after a median of 38 days [IQR: 34–41] since the onset of the index case (Figure 1). Overall, 81 (63%) participants showed evidence of SARS-CoV-2 infection, confirmed either by PCR-testing (*n* = 64, 50%) or positive NAb serology (*n* = 80, 62%) (Supplementary Table 1). Out of 81 COVID-19 cases, four did not experience any symptoms, 73 had experienced mild symptoms, and four required hospital care (Supplementary Table 3).

In five households, one of the other COVID-19 cases had an earlier symptoms onset than the index case, which led to a reclassification of another household member as a primary case and of index cases as secondary cases. In two households, one other case had the same timing of onset as the index case, which led to exclusion of five participants from two households. Hence, we included 124 participants from 37 different households in the household transmission component of the study (Supplementary Table 1). Among these 37 different households, 40 out of 87 household contacts of the primary cases developed a secondary infection confirmed either *via* RT-PCR (*n* = 28, 32%) or positive NAb serology (*n* = 39, 45%) by the end of the household transmission follow-up. In 14/37 (38%) households, no secondary cases were identified. Overall, the SAR was 45% [95% CI: 35–56%] with a median effective reproduction number of 1 [IQR: 0–2] on a household level (Table 1).

**TABLE 1:** Household transmission dynamics.

	Secondary cases (*N* = 40)	Household contacts (*N* = 87)	Percentage of secondary cases	Effective reproduction number
	*N*	*N*	%	Median [IQR]
**Age of the primary case**				
0–9 years old (*n* = 1)	0	3	0%	0
10–19 years old (*n* = 2)	7	7	100%	3.5 [3–4]
20–29 years old (*n* = 4)	6	13	46%	2 [1–2]
30–39 years old (*n* = 9)	6	14	43%	1 [0–1]
40–49 years old (*n* = 15)	14	41	34%	1 [0–1]
≥ 50 years old (*n* = 6)	6	9	67%	1 [0–2]
**Household size**				
2 members (*n* = 11)	7	11	64%	1 [0–1]
3 members (*n* = 8)	5	16	31%	0 [0–1.5]
4 members (*n* = 13)	15	39	38%	1 [0–2]
5 members (*n* = 4)	7	16	44%	1.5 [0.5–3]
6 members (*n* = 1)	5	5	100%	5 [5–5]
**Rooms**				
<1 room per household member	22	52	42%	1 [0–1]
1–2 rooms per household members	15	27	56%	1 [0.5–2]
>2 rooms per household members	2	8	25%	0 [0–1]
**Bedrooms**				
<1 bedroom per household member	35	75	47%	1 [0–2]
At least one bedroom per household members	4	12	33%	0 [0–1]
Total	39	87	45%	1 [0–2]

Out of 39 secondary cases, 88% [95% CI: 74–95%] (*n* = 37) reported at least one symptom, while 58% [95% CI: 43–72%] (26/45) of healthy household contacts reported they had experienced symptoms between primary case onset and the end of follow-up (*p*-value = 0.002). The most reported symptoms among secondary cases were cough (*n* = 26, 67%) and headache (*n* = 25, 69%) (Supplementary Table 2). We did not find any significant differences regarding age, sex, type of relationship to the primary case and previous comorbidities between secondary cases and household contacts who did not get infected.

### Follow-up of SARS-CoV-2 immunity

Out of 80 seroconverted participants, 70 (88%) participated in the 3-month sample collection at a median of 88 days [IQR: 81–99] after recruitment and first sample collection. All participants regardless of seroconversion (*n* = 129) were invited for 6 and 12-month sample collection. At 6 months, 113 participated and 111 donated sera, including 74 cases, during the 6-month sample collection at a median of 185 [IQR: 178–198] days after recruitment, Finally, at 12 months, 101 participants, including 71 cases donated sera at a median of 375 [IQR: 367–383] days post initial recruitment (Figure 1). Between the 6 and 12 months visit, 12 previously seroconverted participants had received Comirnaty (*n* = 8) or Vaxzevria (*n* = 4) COVID-19 vaccine and were only included in the assessment of the persistence of anti-nucleoprotein IgG Ab but excluded from the assessment of NAb and anti-spike IgG Ab persistence at 12 months.

Of the cases, 99% (80/81) had developed NAb, anti-spike and anti-nucleoprotein IgG Ab during the initial follow up (Table 2). One participant did not develop antibodies despite a positive PCR test. NAb persisted in 90% (63/70), 85% (63/74), and 78% (45/58) of the cases 3, 6, and 12 months after infection, respectively (Table 2). Anti-spike IgG Ab persisted at a higher rate compared to NAb, as 100% (69/69), 97% (72/74), and 93% (55/59) of the cases had anti-spike IgG Ab after 3, 6, and 12 months, respectively. Anti-nucleoprotein IgG Ab persisted for a shorter period, with 99% (68/69), 78% (58/74), and 55% (39/71) of cases having anti-nucleoprotein IgG Ab after 3, 6, and 12 months, respectively (Table 2).

**TABLE 2:** Confirmed cases positive for IgG or neutralizing antibodies (NAb) against SARS-CoV-2 during initial follow up and 3–12 months after infection.

		Initial follow up, *n* (%)^a^	Three months convalescent visit, *n* (%)	Six months convalescent visit, *n* (%)	Twelve months convalescent visit, *n* (%)^b^
Anti-nucleoprotein IgG	Positive	80 (99%)	68 (99%)	58 (78%)	39 (55%)
	Negative	1 (1%)	1 (1%)	16 (22%)	32 (45%)
Anti-spike IgG	Positive	80 (99%)	69 (100%)	72 (97%)	55 (93%)
	Negative	1 (1%)	0 (0%)	2 (3%)	4 (7%)
NAb to wild-type SARS-CoV-2	Positive	80 (99%)	63 (90%)	63 (85%)	45 (78%)
	Negative	1 (1%)	4 (6%)	6 (8%)	11 (19%)
	Borderline	0 (0%)	3 (4%)	5 (7%)	2 (3%)

^a^Initial follow up was conducted 7, 14, and 28 days after the onset of the index case. ^b^Anti-spike and NAb results include only subjects who were not vaccinated for COVID-19 before sample collection. Therefore, at 12 months the number of subjects included in the calculation is lower for anti-spike (n = 60) and NAb (n = 59; one additional sample was excluded due to limited sample volume) compared to anti-nucleoprotein (n = 71).

**FIGURE 2:**
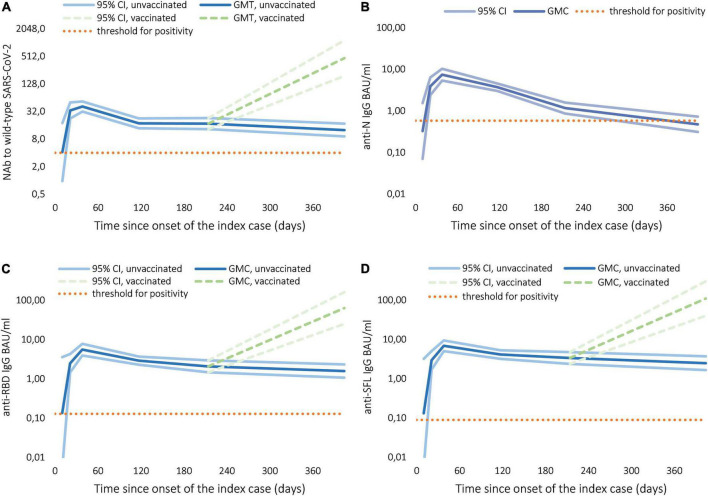
Development and follow-up of IgG and neutralizing antibodies (NAb) to SARS-CoV-2 among confirmed cases, geometric mean concentration (GMC) in binding antibody units (BAU/ml), titer (GMT), and 95% confidence interval (CI). **(A)** NAb to wild-type SARS-CoV-2. **(B)** IgG to wild-type SARS-CoV-2 nucleoprotein (N). **(C)** IgG to wild-type SARS-CoV-2 receptor binding domain (RBD). **(D)** IgG to wild-type SARS-CoV-2 full-length spike glycoprotein (SFL).

In the samples collected from confirmed cases during the initial follow-up, we observed a progressive increase in the mean NAb, anti-spike and anti-nucleoprotein IgG levels, all peaking at the 1-month time point (Figure 2). The decrease over time differed between antibodies produced against SARS-CoV-2 nucleoprotein and spike glycoprotein (Figures 2B–D). NAb and IgG Ab levels to full-length spike glycoprotein (SFL) and receptor binding domain (RBD) decreased notably between 3- and 6-month sampling, but only slightly between 6- and 12-month sampling points (Figures 2C,D and Table 3). One month after infection anti-nucleoprotein IgG Ab levels decreased severely so that at 12 months, almost half of the cases could not be distinguished from non-cases (Figure 2B and Table 3). All 12 subjects that had been vaccinated after the 6-month sample collection had high NAb and anti-spike Ab levels at 12 months (Figures 2A,C,D, indicated with dashed green lines). Anti-spike, nucleoprotein and NAb titers were higher among participants who had required hospital care compared to cases with mild symptoms across all time points (Table 3).

**TABLE 3:** Antibody levels of confirmed cases over 12 months, geometric mean neutralizing antibody titers (GMT) and IgG antibody concentrations (GMC) in binding antibody units (BAU/ml).

			Initial follow-up^a^	Three months convalescent visit	Six months convalescent visit	Twelve months convalescent visit
Anti-nucleoprotein IgG	Asymptomatic cases	GMC [95% CI]	8.5 [0.88–83]	3.7 [0.27–54]	1.7 [0.11–27]	1.2 [0.06–21]
		n	4	3	3	3
	Cases with symptoms	GMC [95% CI]	7.1 [4.8–10]	3.4 [2.7–4.4]	1.1 [0.78–1.5]	0.41 [0.27–0.68]
		n	60	62	67	64
	Cases requiring hospital care	GMC [95% CI]	53 [42–66]	10 [7.5–14]	4.4 [1.9–11]	2.4 [0.92–6.1]
		n	3	4	4	4
Anti-SFL IgG	Asymptomatic cases	GMC [95% CI]	8.5 [5.1–14]	3.2 [0.92–11]	2.8 [0.46–16]	2.1 [0.17–26]
		n	4	3	3	3
	Cases with symptoms	GMC [95% CI]	6.2 [4.1–8.7]	3.9 [3–5.1]	3 [2.1–4.4]	2.3 [1.5–3.7]^b^
		n	60	62	67	53^b^
	Cases requiring hospital care	GMC [95% CI]	65 [15–290]	12 [2.3–65]	17 [6.2–44]	8.1 [2.4–26]^b^
		n	3	4	4	3^b^
Anti-RBD IgG	Asymptomatic cases	GMC [95% CI]	7.6 [3.1–19]	2.7 [0.9–7.2]	1.6 [0.36–7.2]	1.4 [0.17–13]
		n	4	3	3	3
	Cases with symptoms	GMC [95% CI]	5.2 [3.6–7.2]	2.7 [2.2–3.6]	2 [1.3–2.7]	1.4 [1–2.3]^b^
		n	60	62	67	53^b^
	Cases requiring hospital care	GMC [95% CI]	63 [34–120]	6.5 [2.2–19]	11 [5–26]	5.2 [2.7–10]^b^
		n	3	4	4	3^b^
NAb to wild-type SARS-CoV-2	Asymptomatic cases	GMT [95% CI]	83 [32–220]	13 [8.7–20]	17 [2–150]	15 [2.1–110]
		n	4	3	3	3
	Cases with symptoms	GMT [95% CI]	45 [33–60]	17 [13–22]	16 [12–22]	12 [8.4–17]^b^
		n	59	63	67	53^b^
	Cases requiring hospital care	GMT [95% CI]	370 [74–1900]	52 [20–131]	60 [20–180]	25 [9.4–69]^b^
		n	3	4	4	4^b^

SFL, full-length spike glycoprotein; RBD, receptor binding domain; CI, confidence interval.^a^GMC and GMT calculations include the highest antibody level measured during the initial follow up per participant. ^b^GMC and GMT calculations include only the non-vaccinated.

## Discussion

Exposure to an individual with SARS-CoV-2 in a household led to secondary infection in nearly half of the household contacts (SAR 45%). A convalescent sampling of patients showed that NAb to wild-type SARS-CoV-2 persisted in 78% of cases 12 months after infection. Anti-spike IgG Ab persisted in 93% of cases whilst only 55% had anti-nucleoprotein IgG Ab 12 months after infection. The slope at which NAb and anti-spike IgG Ab levels decreased between 3–6 and 6–12 months was stable, implying longevity of antibodies produced against SARS-CoV-2 spike glycoprotein.

Depending on the duration of follow-up and the diagnostic methods, the estimates obtained by previous SARS-CoV-2 household transmission studies are very heterogeneous, with SARs ranging from 4.6 to 89.8% (13–26). In a systematic meta-analysis the average SAR was found to be 19%, with an increase in household transmission observed over time, possibly due to improved diagnostics, longer follow-up and more contagious variants (27). Our findings are in line with other studies where seroconversion was used for the identification of secondary cases over a long duration of follow-up. In the Netherlands, using RT-PCR testing and SARS-CoV-2 IgG enzyme-linked immunosorbent assay (ELISA), Reukers et al. estimated that the overall household SAR was 43% (95% CI: 33–53%) after 4–6 weeks of follow-up (25). While in the United States, when using a CDC-developed SARS-CoV-2 ELISA, Lewis et al. showed that after 14 days of follow-up, the SAR was 29% (95% CI: 23–36%) overall (28). It is plausible that our estimate of the SAR, as ones from previously published studies are underestimated, knowing that not all who are infected will develop detectable antibodies (29) but could still develop T-cell responses (30–32).

Our study suggests long-term persistence of antibodies following infection with mild symptoms. Neutralizing antibodies have been shown to correlate with protection against symptomatic infection (33, 34). However, no specific NAb level that would serve as a serological correlate for protection against SARS-CoV-2 infection or disease has been established. Emerging variants that carry mutations in the RBD of the spike protein, which is a major target of neutralizing antibodies, require considerably higher concentrations of antibodies for efficient neutralization and are thus capable of escaping prior immunity (35–37). In addition, estimations of persistence are heavily affected by the sensitivity and specificity of the antibody test used. To obtain the most realistic estimation of NAb prevalence 1 year after infection, we used a neutralization assay with live virus and a low starting dilution of serum for increased sensitivity. Although the consensus is that NAb titers decline but persist at least 1 year after infection even after mild disease, the estimates of NAb prevalence at this time point are diverse. We have previously reported that NAb to wild-type SARS-CoV-2 persisted in 89% of participants 13 months after infection (38). This slightly higher rate of persistence is likely explained by differing study populations, as the previous study involved more people with severe COVID-19. Other studies using a live SARS-CoV-2 assay have reported measurable NAb in 73% (39) and 85% (40) of participants 9 months and a year after SARS-CoV-2 infection, respectively. Studies performed with a surrogate virus have reported neutralizing activity to persist e.g., 60% 9 months after infection (41) and in 58% (42) and 73% (43) of participants a year after infection.

Although seropositivity and persistence of IgG antibodies are also affected by the assay’s sensitivity and specificity (44), many have reported surprisingly similar, e.g., 94% (45), 95% (46), and 96% (47), positivity rates 6 months after infection. A year after infection estimates of IgG prevalence against SARS-CoV-2 spike range from 58 to 83% (38, 42, 43, 46, 48). Furthermore, we found that between 6 and 12 months, anti-spike IgG concentrations decreased only slightly, implying long-term persistence 1 year after infection. Slowly decreasing anti-spike IgG Ab levels between 6 and 12 months after infection have also been reported elsewhere (46, 49, 50). Here we observed nucleoprotein antibodies to wane more quickly to a level where they were indistinguishable from negative control sera, and similar has been observed by others (45, 48–51).

Our study had several limitations: First, this was not a randomly selected cohort of households as we recruited participants through voluntary convenience sampling. Second, due to the lag between onset and availability of RT-PCR results, most participants were recruited retrospectively, which prevented us from conducting early household visits to estimate serial intervals for all subjects. Additionally, our limited sample size did not allow comparison of secondary transmission rate depending on the age of the primary case and our estimation of the effective reproductive number was influenced by the limited size of households, with a median of 3 [IQR: 2–4] members. However, this was in line with the demographics of Helsinki, where 51% of households consist of at least two members including 21% of households with 3 or more members (52). Finally, we conducted this study at a time before access to immunization and the emergence of variants of concern (53), two parameters that influence SARS-CoV-2 transmission. SAR should be lower among vaccinated contacts (54), however, transmission rate and the impact of vaccination appears to differ between variants (55, 56).

One of the strengths of our study was, that it was conducted using a highly sensitive and specific antibody assay (10). Furthermore, unlike most household transmission studies, we measured not only IgG Ab but also NAb to SARS-CoV-2 and assessed household transmission over a 1-month period, providing enough time for the development of antibodies, in case of secondary infections. Finally, only a few participants were lost to follow-up, allowing us to assess trends in IgG Ab and NAb for 78% of participants over 12 months.

We found that the majority of those that became infected developed long-lasting antibodies to SARS-CoV-2 spike glycoprotein and had neutralizing capabilities at least a year after infection. A 2-year follow-up on this study cohort is underway. However, our results on persisting antibodies should be applied cautiously to the current pandemic situation, as antibodies produced against earlier SARS-CoV-2 lineages provide limited protection against the Omicron variants (57, 58). When living in the same household of an RT-PCR confirmed case, the risk of transmission to naïve or unvaccinated household members is extremely high as 1 out of 2 get infected. The challenge in a household setting is that adherence to infection prevention and control measures is conceivably low. Household members at risk of severe COVID-19, as well as their treating physicians should be informed of this risk, given detailed instructions on how to reduce physical contact with the index case, and followed up clinically. Moreover, consideration should be given to the isolation of the cases in separate facilities, even though viral transmission might have already occurred in other household members before the first diagnosis and symptoms (59).

## Data availability statement

The isolates used in this study can be found on GISAID online repository (https://www.gisaid.org) under the following accession number: EPI_ISL_407079 and EPI_ISL_412971.

## Ethics statement

The Finnish communicable diseases law and the law on the duties of the Finnish Institute for Health and Welfare allowed the implementation of the initial household transmission study and 3 months convalescent sample collection without seeking further institutional ethical review [60, 61].

## Author contributions

TD, HN, NI, MM, and AP conceived and designed the study. TL supervised contact tracing as well as helped in the recruitment process. TD, LH, OL, ML, SV, and HN recruited participants to the study and collected samples and data. NI developed, supervised, and interpreted the virological analysis. PÖ cultivated the viral strains for immunological analyses. CV, AH, AS, NE, KL, HV, HH, and MM developed, performed, supervised, and interpreted the immunological analyses. TD and AS performed the statistical analyses and wrote the first version of the report. All authors critically reviewed and approved the final version of the report.

## Conflict of interest

The Finnish Institute for Health and Welfare had received research funding for studies not related to COVID-19 from GlaxoSmithKline Vaccines (NE, CV, AP, and MM as investigators), Pfizer (AP), and Sanofi Pasteur (AP). The remaining authors declare that the research was conducted in the absence of any commercial or financial relationships that could be construed as a potential conflict of interest.

## Publisher’s note

All claims expressed in this article are solely those of the authors and do not necessarily represent those of their affiliated organizations, or those of the publisher, the editors and the reviewers. Any product that may be evaluated in this article, or claim that may be made by its manufacturer, is not guaranteed or endorsed by the publisher.
