# Supplementary material for: High secondary attack rate and persistence of SARS-CoV-2 antibodies in household transmission study participants, Finland 2020–2021
Source: Front Med (Lausanne). 2022 Jul 28;9:876532. doi: 10.3389/fmed.2022.876532 (PMC9366099; doi:10.3389/fmed.2022.876532)
Supplement: Supplementary file 1 [file Data_Sheet_1.docx]

## ***Supplementary material***

**1 Supplementary figures and tables**

Table S1: Description of household study participants.

|  | Primary cases (n=37) | Household contacts (n=87) | Excluded participants (n=5) |
| --- | --- | --- | --- |
|  | N (%) | N (%) | N (%) |
| **Sex** |  |  |  |
| Female | 18 (49%) | 41 (47%) | 3 (60%) |
| Male | 19 (51%) | 46 (53%) | 2 (40%) |
|  |  |  |  |
| **Age categories** |  |  |  |
| 0-9 years old | 1 (3%) | 15 (17%) | 1 (20%) |
| 10-19 years old | 2 (5%) | 23 (27%) | 0 |
| 20-29 years old | 4 (11%) | 6 (7%) | 0 |
| 30-39 years old | 9 (24%) | 11 (13%) | 4 (80%) |
| 40-49 years old | 15 (41%) | 16 (18%) | 0 |
| ≥50 years old | 6 (16%) | 16 (18%) | 0 |
|  |  |  |  |
| **Pre-existing conditions** |  |  |  |
| None | 29 (78%) | 68 (78%) | 4 (80%) |
| At least one | 8 (22%) | 19 (22%) | 1 (20%) |
| - Obesity | 4 (11%) | 9 (10%) | 0 |
| - Cancer | 0 | 1 (1%) | 0 |
| - Chronic heart condition | 1 (3%) | 1 (1%) | 0 |
| - Asthma | 2 (5%) | 3 (3%) | 0 |
| - Chronic lung condition | 0 | 1 (1%) | 0 |
| - Chronic hematologic condition | 0 | 1 (1%) | 0 |
| - Chronic neurologic condition | 3 (8%) | 3 (3%) | 1 (20%) |
| - Other condition | 4 (11%) | 7 (8%) | 0 |
|  |  |  |  |
| **Reported symptoms** |  |  |  |
| None | 0 | 24 (28%) | 0 |
| At least one | 37 (100%) | 63 (72%) | 5 (100%) |
| - Fever | 23 (62%) | 22 (25%) | 4 (80%) |
| - Sore throat | 20 (54%) | 30 (35%) | 2 (40%) |
| - Cough | 29 (78%) | 34 (39%) | 4 (80%) |
| - Runny nose | 13 (35%) | 32 (37%) | 5 (100%) |
| - Shortness of breath | 13 (35%) | 13 (15%) | 3 (60%) |
| - Chills | 26 (72%) | 23 (27%) | 5 (100%) |
| - Vomiting | 3 (9%) | 3 (4%) | 0 |
| - Nausea | 11 (31%) | 15 (18%) | 0 |
| - Diarrhoea | 13 (38%) | 19 (23%) | 2 (40%) |
| - Headache | 23 (70%) | 38 (46%) | 2 (40%) |
| - Rash | 3 (10%) | 3 (4%) | 0 |
| - Conjunctivitis | 0 | 2 (2%) | 0 |
| - Myalgia | 28 (80%) | 22 (27%) | 2 (40%) |
| - Arthralgia | 16 (48%) | 17 (21%) | 2 (40%) |
| - Loss of appetite | 20 (57%) | 17 (20%) | 0 |
| - Nosebleed | 3 (9%) | 7 (8%) | 3 (75%) |
| - Fatigue | 35 (95%) | 38 (44%) | 4 (80%) |
| - General malaise | 21 (58%) | 14 (17%) | 1 (20%) |
| - Altered consciousness | 3 (9%) | 2 (2%) | 0 |
| - Anosmia | 23 (66%) | 17 (21%) | 4 (80%) |
| Hospital care requirement | 4 (11%) | 0 | 0 |
|  |  |  |  |
| **Positive diagnosis methods** |  |  |  |
| - Positive SARS-CoV-2 RT-PCR | 33 (89%) | 27 (31%) | 4 (80%) |
| - SARS-CoV-2 neutralizing antibodies | 36 (97%) | 39 (45%) | 5 (100%) |

Table S2: Secondary cases and healthy household contacts characteristics and symptoms.

|  | Healthy household contacts (N=48) | Secondary cases (N=39) | p-value |
| --- | --- | --- | --- |
|  | N (%) | N (%) |  |
| Sex |  |  |  |
| Female | 20 (42%) | 21 (54%) | 0.258 |
| Male | 28 (58%) | 18 (46%) |  |
|  |  |  |  |
| Age groups |  |  |  |
| 0-9 years old | 10 (21%) | 5 (13%) | 0.808 |
| 10-19 years old | 11 (23%) | 12 (31%) |  |
| 20-29 years old | 4 (8%) | 2 (5%) |  |
| 30-39 years old | 7 (15%) | 4 (10%) |  |
| 40-49 years old | 8 (17%) | 8 (21%) |  |
| ≥50 years old | 8 (17%) | 8 (21%) |  |
|  |  |  |  |
| **Relationship to the primary case** |  |  |  |
| Child | 21 (44%) | 13 (33%) | 0.801 |
| Parent/Stepparent | 9 (19%) | 9 (23%) |  |
| Spouse/partner | 14 (29%) | 13 (33%) |  |
| Sibling | 4 (8%) | 4 (10%) |  |
|  |  |  |  |
| **Positive diagnosis methods** |  |  |  |
| Positive PCR-testing | 0 | 27 (69%) |  |
| SARS-CoV-2 neutralizing antibodies | 0 | 39 (100%) |  |
| IgG antibodies to SARS-CoV-2 nucleoprotein | 3 (6%) | 39 (100%) |  |
|  |  |  |  |
| **Pre-existing conditions** |  |  |  |
| None | 38 (79%) | 30 (77%) | 0.801 |
| At least one | 10 (21%) | 9 (23%) |  |
| - Obesity | 5 (11%) | 4 (10%) | 1.000 |
| - Cancer | 1 (2%) | 0 | 1.000 |
| - Chronic heart condition | 1 (2%) | 0 | 1.000 |
| - Asthma | 3 (6%) | 0 | 0.249 |
| - Chronic lung condition | 0 | 1 (3%) | 0.448 |
| - Chronic hematologic condition | 1 (2%) | 0 | 1.000 |
| - Chronic neurologic condition | 2 (4%) | 1 (3%) | 1.000 |
| - Other conditions | 2 (4%) | 5 (13%) | 0.239 |
|  |  |  |  |
| **Reported symptoms** |  |  |  |
| None | 20 (42%) | 4 (10%) | 0.001 |
| At least one | 28 (58%) | 35 (90%) |  |
| - Fever | 3 (6%) | 19 (49%) | 0.000 |
| - Sore throat | 11 (23%) | 19 (49%) | 0.012 |
| - Cough | 8 (17%) | 26 (67%) | 0.000 |
| - Runny nose | 12 (25%) | 20 (51%) | 0.011 |
| - Shortness of breath | 3 (6%) | 10 (26%) | 0.012 |
| - Chills | 7 (15%) | 16 (44%) | 0.002 |
| - Vomiting | 1 (2%) | 2 (5%) | 0.411 |
| - Nausea | 5 (11%) | 10 (29%) | 0.038 |
| - Diarrhoea | 6 (13%) | 13 (35%) | 0.015 |
| - Headache | 13 (28%) | 25 (69%) | 0.000 |
| - Rash | 1 (2%) | 2 (5%) | 0.577 |
| - Conjunctivitis | 0 | 2 (5%) | 0.187 |
| - Myalgia | 4 (9%) | 18 (51%) | 0.000 |
| - Arthralgia | 4 (9%) | 13 (36%) | 0.002 |
| - Loss of appetite | 4 (9%) | 13 (34%) | 0.004 |
| - Nosebleed | 4 (8%) | 3 (8%) | 1.000 |
| - Fatigue | 6 (13%) | 13 (35%) | 0.015 |
| - General malaise | 6 (13%) | 8 (22%) | 0.273 |
| - Altered consciousness | 1 (2%) | 1 (3%) | 1.000 |
| - Anosmia | 0 | 17 (47%) | 0.000 |
| Hospital care requirement | 0 | 0 | 1.000 |

Table S3: COVID-19 cases characteristics and symptoms.

|  | COVID-19 cases (N=81) |
| --- | --- |
|  | N (%) |
| Sex |  |
| Female | 42 (52%) |
| Male | 39 (48%) |
|  |  |
| Age groups |  |
| 0-9 years old | 7 (9%) |
| 10-19 years old | 14 (17%) |
| 20-29 years old | 6 (7%) |
| 30-39 years old | 17 (21%) |
| 40-49 years old | 23 (28%) |
| ≥50 years old | 14 (17%) |
|  |  |
| **Positive diagnosis methods** |  |
| Positive PCR-testing | 64 (79%) |
| SARS-CoV-2 neutralizing antibodies | 80 (99%) |
| IgG antibodies to SARS-CoV-2 nucleoprotein | 80 (99%) |
|  |  |
| **Pre-existing conditions** |  |
| None | 63 (78%) |
| At least one | 18 (22%) |
| - Obesity | 8 (10%) |
| - Cancer | 0 |
| - Chronic heart condition | 1 (1%) |
| - Asthma | 2 (2%) |
| - Chronic lung condition | 1 (1%) |
| - Chronic hematologic condition | 0 |
| - Chronic neurologic condition | 5 (6%) |
| - Other conditions | 9 (11%) |
|  |  |
| **Reported symptoms** |  |
| None | 4 (5%) |
| At least one | 77 (95%) |
| - Fever | 46 (57%) |
| - Sore throat | 41 (51%) |
| - Cough | 59 (73%) |
| - Runny nose | 38 (47%) |
| - Shortness of breath | 26 (32%) |
| - Chills | 47 (61%) |
| - Vomiting | 5 (7%) |
| - Nausea | 21 (28%) |
| - Diarrhoea | 28 (37%) |
| - Headache | 50 (68%) |
| - Rash | 5 (7%) |
| - Conjunctivitis | 2 (3%) |
| - Myalgia | 48 (64%) |
| - Arthralgia | 31 (42%) |
| - Loss of appetite | 33 (43%) |
| - Nosebleed | 9 (12%) |
| - Fatigue | 64 (79%) |
| - General malaise | 30 (39%) |
| - Altered consciousness | 4 (5%) |
| - Anosmia | 44 (58%) |
| Hospital care requirement | 4 (5%) |
